# Evaluation of Mechanical and Microstructural Properties and Global Warming Potential of Green Concrete with Wheat Straw Ash and Silica Fume

**DOI:** 10.3390/ma15093177

**Published:** 2022-04-27

**Authors:** Kaffayatullah Khan, Muhammad Ishfaq, Muhammad Nasir Amin, Khan Shahzada, Nauman Wahab, Muhammad Iftikhar Faraz

**Affiliations:** 1 Department of Civil and Environmental Engineering, College of Engineering, King Faisal University, Al-Ahsa 31982, Saudi Arabia; kkhan@kfu.edu.sa; 2 Al Bilad Bank Scholarly Chair for Food Security in Saudi Arabia, The Deanship of Scientific Research, The Vice Presidency for Graduate Studies and Scientific Research, King Faisal University, Al-Ahsa 31982, Saudi Arabia; mfaraz@kfu.edu.sa; 3 Department of Civil Engineering, University of Engineering and Technology, Peshawar 25120, Pakistan; mishfaq948@gmail.com (M.I.); khanshahzada@uetpeshawar.edu.pk (K.S.); 4 Department of Civil and Environmental Engineering, University of Rome “La Sapienza”, Via Eudossiana 18, 00184 Rome, Italy; nauman.wahab@uniroma1.it; 5 Department of Mechanical Engineering, College of Engineering, King Faisal University, Al-Ahsa 31982, Saudi Arabia

**Keywords:** wheat straw ash, global warming potential, compressive strength, water absorption, microstructure and pore structure

## Abstract

Cement and concrete are among the major contributors to CO_2_ emissions in modern society. Researchers have been investigating the possibility of replacing cement with industrial waste in concrete production to reduce its environmental impact. Therefore, the focus of this paper is on the effective use of wheat straw ash (WSA) together with silica fume (SF) as a cement substitute to produce high-performance and sustainable concrete. Different binary and ternary mixes containing WSA and SF were investigated for their mechanical and microstructural properties and global warming potential (GWP). The current results indicated that the binary and ternary mixes containing, respectively, 20% WSA (WSA20) and 33% WSA together with 7% SF (WSA33SF7) exhibited higher strengths than that of control mix and other binary and ternary mixes. The comparative lower apparent porosity and water absorption values of WSA20 and WSA33SF7 among all mixes also validated the findings of their higher strength results. Moreover, SEM–EDS and FTIR analyses has revealed the presence of dense and compact microstructure, which are mostly caused by formation of high-density calcium silicate hydrate (C-S-H) and calcium hydroxide (C-H) phases in both blends. FTIR and TGA analyses also revealed a reduction in the portlandite phase in these mixes, causing densification of microstructures and pores. Additionally, N_2_ adsorption isotherm analysis demonstrates that the pore structure of these mixes has been densified as evidenced by a reduction in intruded volume and a rise in BET surface area. Furthermore, both mixes had lower CO_2_-eq intensity per MPa as compared to control, which indicates their significant impact on producing green concretes through their reduced GWPs. Thus, this research shows that WSA alone or its blend with SF can be considered as a source of revenue for the concrete industry for developing high-performance and sustainable concretes.

## 1. Introduction

Concrete is a heterogeneous material formulated from natural and synthetic components, combined with water, and forms a versatile, durable, and essential building material. Worldwide, concrete production has significantly increased due to urbanization and urban development, reaching 25 billion tons per year [1]. Subsequently, the yearly production of cement has also soared to 4.1 billion tons globally [2]. Besides its usefulness, cement and concrete production has resulted in massive carbon emissions as well. Apart from the carbon emissions, the usage of raw materials in cement production is also responsible for the depletion of natural resources and its associated carbon footprint [3]. Hence, the annual manufactured carbon emissions from the cement industry are more than 5% of the total global anthropogenic emissions [4]. This vast amount of CO_2_ generation is also a leading factor in creating the issues related to global warming and climate change.

These issues can be controlled by following the sustainability approach in the construction sector. One such approach is to replace cement with supplementary cementitious materials (SCMs) at the construction site or during cement manufacturing. Another approach is to conserve natural resources by replacing aggregates with alternate ecofriendly materials. Hence, the search for supplementary cementitious materials has resulted in the usage of industrial byproducts such as volcanic ash [5,6], silica fume (SF) [7,8], fly ash [9,10], electric arc furnace slag [11,12], tire ash [13,14], copper slag [15,16], etc., and agricultural byproducts such as sugarcane bagasse ash [17,18], rice husk ash [19,20], wood-waste ash [21,22], wheat straw ash (WSA) [23,24], and others. The utilization of these agroindustrial wastes has been proved to be promising both as supplementary cementitious material to replace cement and/or fine aggregates [24]. Furthermore, the usage of these byproducts offers many advantages such as improved mechanical and durability properties, reduction in waste generation, reduction in cost by replacing cement/aggregates, and most importantly, the decrease in carbon emissions [25,26]. Therefore, Luhar et al. [4] reviewed the usage of agriculture waste in concrete. They concluded that agrowaste could be used as an effective supplementary cementitious material in concrete, resulting in the development of green concrete. Rattanachu et al. [27] observed that 20% replacement of OPC with finely ground rice husk ash can significantly improve the compressive strength of concrete. In addition, the utilization of SCMs also improves the durability of concrete [2]. In another study, Rashad [3] replaced fine aggregate with metakaolin at 10%, 20%, 30%, 40%, and 50% by weight. The experimental study revealed increased splitting tensile strength, compressive strength, and abrasion resistance of metakaolin mixed concrete at 40% replacement.

In developing countries, agrowaste is one of the significant issues which arise from different food crops such as sugarcane, rice, and wheat. Wheat is one of the most important cereal crops and a major food source for around 2.5 billion people globally [28]. The worldwide wheat production was estimated to be 750 million tons from 2016 to 2017 [29]. Pakistan ranked high in the wheat-producing countries around the globe. In the year 2017–2018, Pakistan produced about 26.6 million tons of wheat. In the Gulf region, the Kingdom of Saudi Arabia is known as a major wheat-producing country. The annual wheat production of the Kingdom of Saudi Arabia is estimated at 700,000 tons [30]. Pan and Sano [31] stated that one kg of wheat grain yields around 1.3 kg wheat straw. Primarily, the wheat straw was utilized to feed cattle. However, open field burning is also practiced in some cases, which causes air pollution (smog) and health issues such as respiratory diseases in that region.

Burned wheat straw produces ash, which is pozzolanic in nature. However, the pozzolanic efficiency of the WSA mainly depends on its source; therefore, its composition varies with regions primarily due to soil properties and climatic conditions [32]. In addition, the burning temperature, exposure time, and particle sizes also play a vital role in its pozzolanic behavior. Biricik et al. [33] reported that wheat straw burned for 5 h at 570 and 670 °C resulted in high-quality WSA. However, WSA produced at a heating temperature of 670 °C showed superior pozzolanic properties. Similarly, an increase in silica content with an increase in burning temperature was also recorded by Amin et al. [29]. Memon et al. [34] investigated the effects of various burning temperatures (500, 600, 700, and 800 °C) on the pozzolanic efficacy of WSA. They observed the transition of amorphous silica into crystalline form with an increasing temperature beyond 600 °C. Therefore, several researchers observed favorable temperatures, which ranged between 570 and 670 °C for an improved WSA production.

Research studies have highlighted the efficacy of the WSA both as filling and pozzolanic material in concrete and mortar. The addition of WSA in mortar increased the compressive and flexural strength [35]. Moreover, the improvement in the mechanical properties of cement mortar was attributed to its filling ability. Furthermore, the addition of WSA in cement mortar or concrete also improves its durability. Qudoos et al. [36] noted that extensively ground WSA exhibited higher compressive strength at all ages than control specimen with 20% cement replacement. Similarly, Amin et al. [29] also observed that 15–20% cement replacement with WSA showed significant enhancement in the strength and ductility of concrete samples at 91 days. WSA as cement or sand replacement decreased the water adsorption and increased the resistance against acid and sulfate attacks. Al-Akhras [37] used WSA as cement replacement up to 15% by weight and concluded that concrete containing WSA showed better resistance to freeze and thaw damage than control specimens. In addition to its standalone performance as a pozzolanic material, the behavior of WSA was also evaluated as a binary mix with other pozzolanic materials such as metakaolin, fly ash, bentonite clay, millet husk ash, and others [3,26,32,38]. The usage of bentonite clay along with WSA was effective in consuming the free lime. Moreover, it improved the resistance of the cementitious matrix against acid attack [38]. The effectiveness of WSA and millet husk ash (MHA) combined was evaluated by Bheel et al. [39]. The results showed improvement in the flexural, tensile, and compressive strength of the specimens containing 15% MHA and 30% WSA combined.

The binary and ternary blends of various agroindustrial wastes have increased its pozzolanic activity. Among many, SF is widely used component of ternary blends to enhance mechanical as well as durability performance of concrete. Generally, SF is obtained as a byproduct during the production of silicon and ferrosilicon alloys at a very high temperature around 2000 °C in an electric furnace arc [40]. Oxygen is eliminated by heating highly pure quartz with coke or coal. An ultrafine powder is obtained, having high porosity and specific surface area in which the silica ranged between 85% and 95% [41]. SF is most commonly used in concrete as a dry, densified form consisting of agglomerates of size from 10 microns to several millimeters. These agglomerates may only partially disintegrate during normal concrete mixing [42]. In order to disperse SF effectively, sonification techniques [43] or the Holland method [44] of mixing SF concrete in a laboratory mixer are necessary for improving the microstructure and pore size of the materials. Murthi et al. [45] presented the effects of OPC, bagasse ash, and nanosilica on the fresh and hardened properties of high-performance concrete. The experimental investigation showed that the addition of nanosilica significantly enhanced the early age strength; however, it reduced the setting time. SF is a highly siliceous material, and its addition to the cement matrix increases the formation of calcium silicate hydrate (C-S-H) gel, resulting in a denser microstructure of the cementitious system. The cement replacement with 10% SF and 20% WSA in lightweight concrete improved its density and strength for structural applications [46]. The microstructure investigation of cementitious matrix containing a ternary blend of OPC, SF, and WSA indicated better mechanical and durability performance than the control specimen [47]. Based on previous research, it has been highlighted that the usefulness of the SCMs increases with the addition of SF. SF, being one of the most reactive siliceous materials, could accelerate the pozzolanic activity inside the cementitious system when used in combination with agro-industrial ashes. Despite several studies, it has been noted that the existing literature is limited to the use of WSA as an individual SCM with low replacement levels.

The purpose of this study is to investigate the novel use of WSA and its blend with SF as a cement substitute to produce environmentally sustainable concrete that does not compromise on its mechanical properties. Therefore, the impact of WSA and its blend with SF, as a high-volume replacement of cement, on the mechanical, durability, and microstructural characteristics of concrete were investigated. Additionally, X-ray fluorescence (XRF) and X-ray diffraction (XRD) methods were used to measure the physical and chemical properties of cement, WSA, and SF. Concrete specimens were prepared, namely, control (100% cement), three binary with only WSA (C/WSA: 90/10, 80/20, and 70/30), and three ternary containing WSA along with SF (C/WSA/SF: 70/25/5, 60/33/7, and 50/40/10). In ternary mixes, a large amount of cement was substituted (up to 50%). Mechanical properties, such as the compressive and split tensile strengths with aging (7, 28, and 91 days), and water absorption (WA) and apparent porosity (AP) after aging for 91 days were evaluated for hardened concrete samples. Additionally, the effect of WSA and its blend with SF on the microstructure and pore structure of the cement paste matrix was studied by employing scanning electron microscopy/energy-dispersive X-ray spectroscopy (SEM–EDS), Fourier transform infrared (FTIR) spectroscopy, thermogravimetric analysis (TGA), and nitrogen (N_2_) adsorption isotherm analysis. At the end, the global warming potential (GWP) of all the concrete mixes was calculated in kg CO_2_-equivalent per unit concrete and kg CO_2_-equivalent per unit concrete/MPa by using the green concrete lifecycle assessment (LCA) tool.

## 2. Materials and Methods

### 2.1. Materials

The main binders used in this study were conventional OPC, consistent with ASTM C150, and commercially available SF and WSA (processed in the laboratory). The specified physical and chemical properties of these materials are listed in Table 1.

Besides binder materials, aggregates from local sources were obtained for using as fillers in concrete. Table 2 shows the sieve analysis results for fine and coarse aggregates. Blending percentages for coarse aggregates are 50% (20 mm down) and 50% (10 mm down). In accordance with ASTM C33, fine aggregate possessed specific gravity of 2.60 and water absorption of 1.03%, while its fineness modulus was 2.54 (Table 2). Specifically, the coarse aggregates had a specific gravity of 2.65 and water absorption of 0.82%, respectively.

#### 2.1.1. Burning and Grinding of Wheat Straw Ash

**Figure 1:**
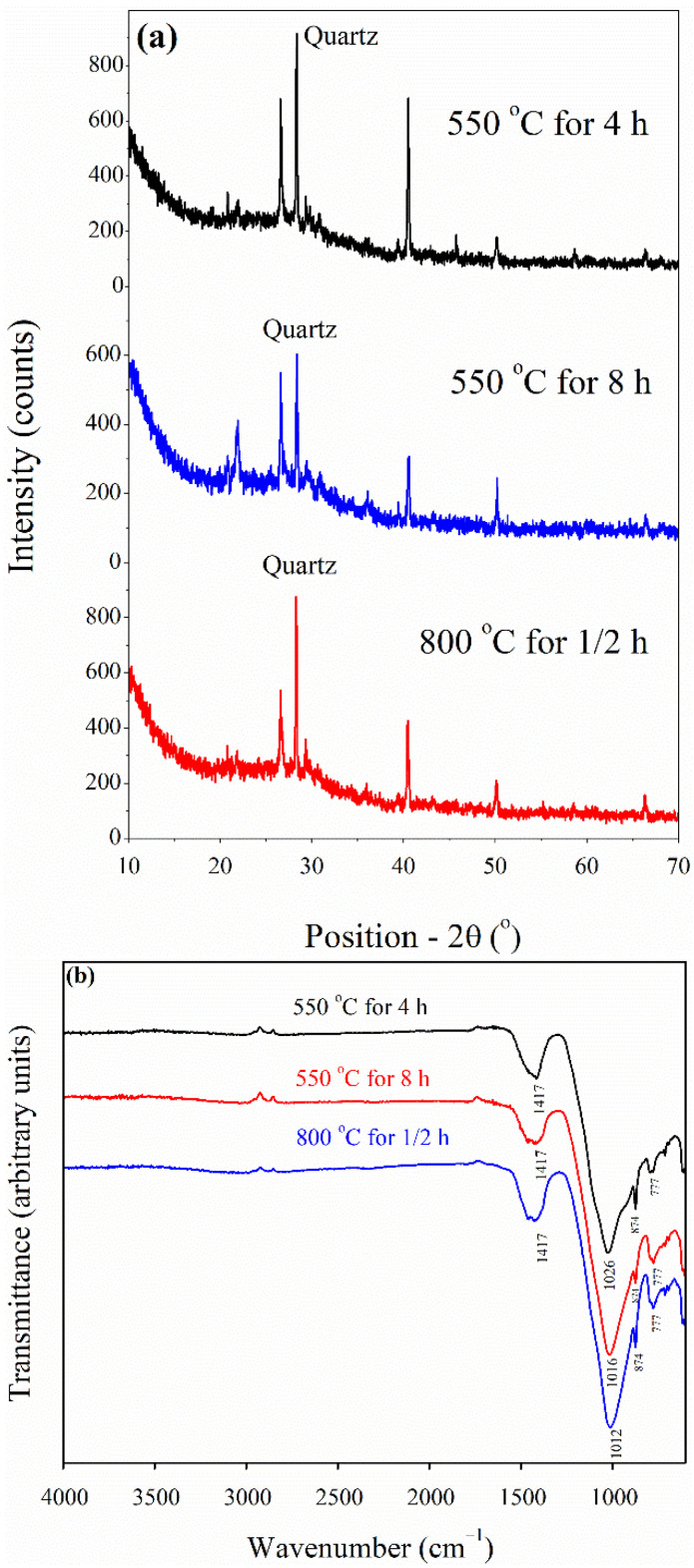
(**a**) XRD pattern and (**b**) FTIR of WSA after heat treatment at 550 °C for 4 and 8 h, and at 800 °C for 30 min.

The chemical properties of WSA are largely dependent on the source (wheat straw), the organic composition, and the sintering temperature [20]. Further, climatic conditions the chemical composition of WSA is influenced by both climatic conditions and geographical location [48]. The WSA obtained was burned at control temperature at 550 for 4 and 8 h and finally at 800 °C for 30 min in a kiln. Each of these samples exposed to different elevated temperatures were subjected to analysis using XRF, XRD, and FTIR to determine their chemical and mineralogical properties. Accordingly, XRD and FTIR results revealed that the maximum amount of amorphous silica was achieved for the sample when burned at 550 °C for 4 h (Figure 1). Based on chemical composition, FTIR, and XRD analyses, the WSA obtained after burning at 550 °C for 4 h was ground to obtain a fine powder. After cooling under normal air, the WSA was ground in a rotary mill for 12 h at 15 rpm. Using this ground WSA, binary concrete specimens containing only WSA and ternary concrete specimens having blends of WSA with SF were prepared in order to evaluate its effectiveness as a partial substitute of cement. The physical and chemical properties of WSA are listed in Table 1.

#### 2.1.2. Concrete Mixture Proportions

In addition to CC, three binary and three ternary concrete mixture proportions were designed. The CC having 100% cement, whereas the binary concrete mixtures were designed by partial substitution of cement with 10%, 20%, and 30% WSA, and are respectively identified as WSA10, WSA20, and WSA30. The ternary mixtures incorporating blends of WSA and SF were designed by substituting high amounts of cement ranging from 30% to 50% as 25% WSA + 5% SF (WSA25SF5), 33% WSA + 7% SF (WSA33SF7), and 40% WSA + 10% SF (WSA40SF10). Table 3 shows the corresponding ingredients of each concrete mixture and the related details of test specimens.

Adding SF to ternary concrete mixtures was primarily to increase the percent of cement replaced by WSA without compromising the mechanical or durability characteristics. Thus, to improve the mechanical strength of binary concrete as compared to those of CC, SF is added at a rate of 5%, 7%, and 10% to concrete mixtures containing a high percentage of WSA as 25%, 33%, and 40%, respectively. The water-to-binder ratio was maintained at 0.35 for all the concrete mixtures. In accordance with the constant water-to-binder ratio and binder content (457 kg/m^3^), the water content for the concrete mixtures was maintained at 160 kg/m^3^. Consequently, to achieve the target workability corresponding to slump values of 120 ± 30 mm, additional dosages (wt.%) of the naphthalene-based water-reducing admixture were employed for each concrete mixture, as listed in Table 3.

### 2.2. Methods

#### 2.2.1. Concrete Mixing and Preparation of Concrete Specimens

Concrete ingredients were mixed using a rotating pan mixer driven by power, following ASTM C192 guidelines. As soon as the required slump was achieved, cylindrical specimens measuring 100 mm in diameter and 200 mm in height were poured with fresh concrete according to ASTM C39. A total of 18 specimens were cast for each concrete mixture to determine the evolution of compressive and splitting tensile strengths after 7, 28, and 91 days of aging (3 identical specimens at each age). The cylindrical molds were covered with plastic sheets after fabrication and kept under standard laboratory temperature of 20 ± 1 °C and relative humidity of 60 ± 5% for 24 h. Concrete specimens were demolded 24 h after casting and moist cured in a curing tub until being tested at 7, 28, and 91 days. The upper and lower surfaces of concrete specimens were leveled by an end-surface grinder after reaching the desired curing period.

#### 2.2.2. Compressive and Splitting Tensile Strength Tests

As per ASTM C39, compression strength tests were conducted on cylindrical specimens using a 200-ton-capacity universal testing machine (UTM, SHIMADZU, Kyoto, Japan) assembly at 0.2 MPa/s loading rate. Additionally, the split tensile test was performed in accordance with ASTM C496 by using a 200-ton UTM at a loading rate of 1 MPa/min.

#### 2.2.3. Water Absorption

In addition to compressive and splitting tensile tests, tests for WA were also performed on hardened concrete samples of all the concrete mixtures according to ASTM C948. To perform WA tests, 100 mm diameter and 50 mm thick concrete samples were cut from 91-day moist-cured concrete specimens. Samples of cut concrete were soaked in water at 21 °C for 24 h and then periodically weighed until the saturated-surface dry weight (SSD) stabilized. If less than 0.5% difference in weight occurred between successive SSD weight measurements, the weight was considered stable. The letter “B” refers to the most recent yielded weight of the samples. Afterward, the mass of the specimens suspended in water was measured to the nearest 0.01 g and labeled as “A”. Concrete samples were oven-dried at 100−110 °C, and their weights were taken every 24 h. Upon achieving a weight loss of <0.5% of the last measured weight, the sample was cooled inside a vacuum desiccator at room temperature and weighed, which is referred to as “C”. To calculate WA and AP, the following equations were used:

Water Absorption (%) = (B − C)/C × 100(1)

Apparent Porosity (%) = (B − C)/(B − A) × 100(2)

#### 2.2.4. Casting and Curing of Paste Samples for Microstructure and Pore Structure Analysis

Paste samples for the control (100% cement) and other mixes with various amounts of WSA (binary) and blends of WSA with SF (ternary), as partial substitutes for cement (weight percentage), were prepared. Using a Hobart mixer (Hobart, IN, USA), a paste of standard consistency was obtained for all mixes. The freshly mixed cement paste was poured into small plastic containers (20 mm diameter and 50 mm height) immediately after mixing. The plastic containers were subsequently sealed and capped prior to curing. Following 91 days in the cured state, the specimens were dried with a solvent exchange method to inhibit the hydration process. Finally, the flaky slices and powder specimens were then washed with isopropanol for 15 min. In order to remove the isopropanol present in the paste samples, the samples were dried in an oven at 40 °C for 30 min. The samples were then sealed in plastic bags for storage before testing.

##### Scanning Electron Microscopy/Energy-Dispersive X-ray Spectroscopy (SEM–EDS) Analysis

The SEM–EDS analysis of all concrete mixes was performed on hardened fragments in the form of slices of cement paste using a JSM-IT100 scanning electron microscope. Isopropanol was used to dry the fragments of hardened paste using the solvent exchange method. After that, all specimens were examined for changes in morphology and composition.

##### Nitrogen Adsorption Isotherm Technique

In addition to the SEM–EDS study, N_2_ sorptiometry was conducted on all the tested concrete mixes. The surface area and pores of the powdered specimen (weighing approximately 0.3 g) obtained from the 91-day cured hardened paste were measured by N_2_ sorption analysis (NOVA2200e, Quanta chrome, Boynton Beach, FL, USA) at 273 K. First, the samples were degassed to remove airborne contaminants that had absorbed during curing. Afterward, N_2_ adsorption was performed at ambient temperatures and controlled pressure on the specimens.

##### Fourier Transform Infrared (FTIR) Analysis

All the mixes studied were additionally characterized using FTIR using a PerkinElmer Spectrum Two FTIR spectrometer to determine their individual phases. The powdered samples of all of the studied mixes were dried and examined under an infrared light source, and the IR spectra were taken in the wavenumber range 400 to 4000 cm^−1^.

##### Thermogravimetric Analysis (TGA) of Cement Pastes

To perform TGA of all the concrete mixes, the powdered specimens obtained from 91-day cured pastes were placed in ceramic vessels fitted with thermogravimetric analyzers. The specimens were heated inside the thermal gravimetric analyzer to a temperature of 20 to 1000 °C at a rate of 10 °C/min, using N_2_ as a medium under static conditions. Moreover, alumina powder was used as a reference for thermal stability at elevated temperatures. Finally, a comparison plot was developed using the built-in software to show the loss of weight of various paste specimens in various temperature ranges.

#### 2.2.5. Methodology to Calculate Global Warming Potential (kg CO_2_-eq) of Concrete Mixes

In this study, the GWP of all concrete mixes was calculated as equivalent to kg CO_2_ (CO_2_-eq) emissions using the green concrete LCA tool. This tool is typically designed to calculate the environmental impact of concrete, its constituent materials (including cement, aggregates, admixtures, and SCMs), and consumption of fuels and water. The environmental carbon footprint effect of all the seven concrete mixes used in this study was evaluated [49]. The study focuses on the major processes associated with raw materials extraction and production. A summary of the assumptions used for the different production technologies, geographic locations, distances, modes of transport, and material types is given in Table 4. Table 5 shows the percentages of power sources in the local electricity grid mix [50].

## 3. Results and Discussion

### 3.1. Compressive and Tensile Strength Evolution of Binary (C/WSA) and Ternary (C/WSA/SF) Concretes

**Figure 2:**
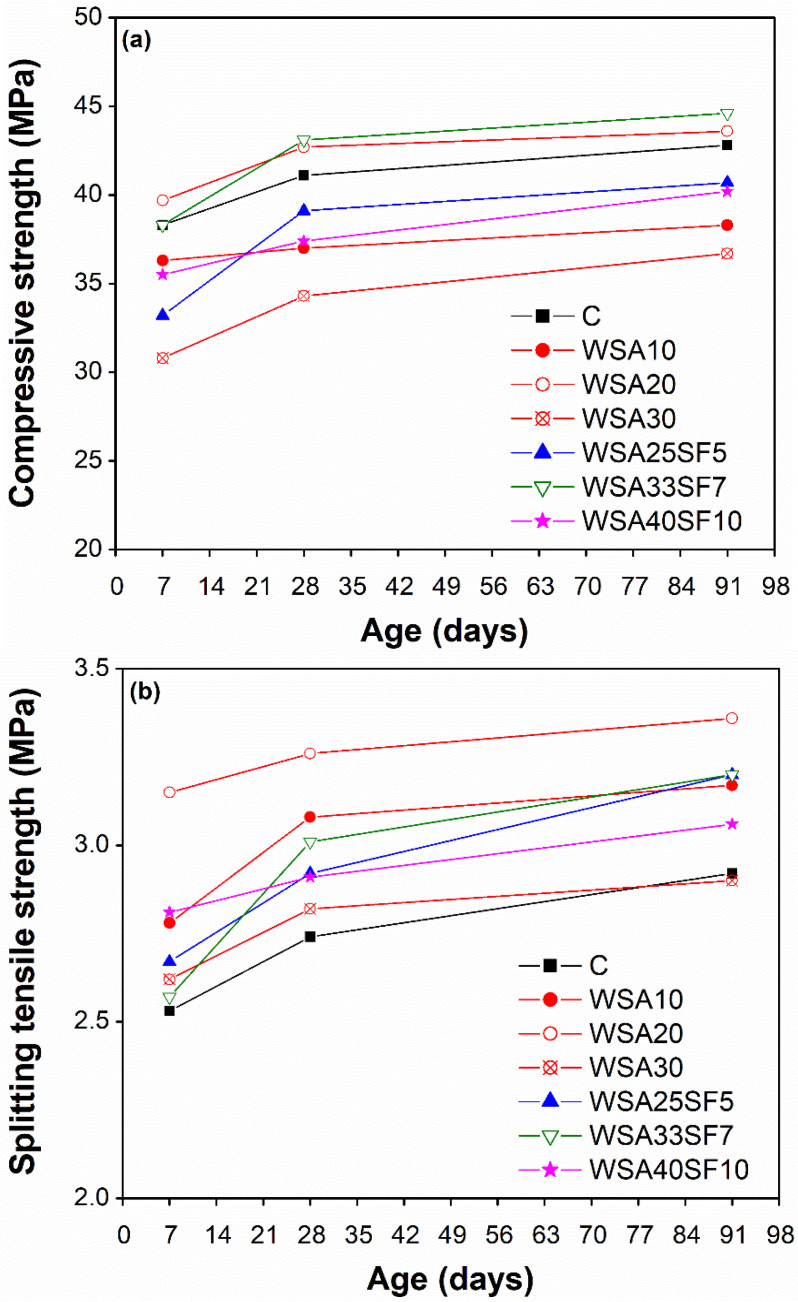
Comparison of strengths of control concrete and concrete having different percentages of WSA alone and blends of WSA with SF: (**a**) compressive strength and (**b**) splitting tensile strength.

A comparison of compressive strength development between CC and those containing WSA alone (binary mixes) and WSA jointly with SF (ternary mixes) is presented in Figure 2a. As listed in Table 3, a relatively low cement substitution rate in binary concrete mixes was set as 10%, 20%, and 30%, whereas in ternary concrete mixes a slightly higher cement replacement was used as 30%, 40%, and 50% owing to highly reactive SF. The purpose of adding different percentage of SF (5%, 7%, and 10%) in combination with different percentages of WSA (25%, 33%, and 40%) was to explore the optimum cement replacement without affecting the strength and durability properties of concrete as compared to control and binary concrete mixes. In addition to technical benefits in terms of mechanical properties, other aspects of blended concrete having WSA with SF in lowering the GWP were also evaluated and compared to those of control and binary concretes. To avoid the effects of external factors, the specimens of all the concrete mixtures studied were water cured under uniform temperature conditions of 20 °C until the age of testing.

As shown in Figure 2a, the binary concrete WSA20 demonstrated highest compressive strength evolution among binary mixes at all testing ages, including the CC. However, a reduction in compressive strength was observed for other binary mixes (WSA10 and WSA30) as compared to CC, regardless of aging. From the current results, it can be seen that the rate and the reduction in compressive strength was higher in WSA30 as compared to that of WSA10. The low strength of WSA10 than that of CC is due to its lower degree of pozzolanic activity, whereas the significantly low compressive strength of WSA30 is due to addition of high amount of WSA, which consequently affected the pozzolanic activity in a significant manner. These results suggested addition of 20% WSA as an optimum amount without compromising the compressive strength of concrete.

To achieve high sustainability in terms of lesser GWP, efforts were made to regain the reduction in the compressive strength of binary mixes containing high percentages of WSA by adding different percentages of SF (5%, 7%, and 10%). The test results show significant improvement in strength of ternary mix with an equal percentage of cement substitution (WSA25SF5) to that of the corresponding binary mix having WSA alone (WSA30) at all ages. Owing to addition of 5% SF, the improvement in strength that occurred remained slightly lower than that of CC. However, an encouraging response was noticed for ternary mix (WSA33SF7) containing a slightly high percentages of SF (7%) in presence of high percentage of WSA (33%), where the compressive strength was higher than that of CC at all ages. These results demonstrated fast early-age hydration and better packing and filling abilities due to slightly increased amount of very fine SF along with the later-age pozzolanic reaction of high-volume WSA. With a further increase in cement substitution with 10% SF in presence of 40% WSA (WSA40SF10), a slight reduction in strength was observed as compared to CC at all ages. This is because a high percentage of cement substitution (50%) affected both the early hydration and later-age pozzolanic reaction due to the production of less calcium hydroxide (C-H). However, the strength performance of this ternary mix (WSA40SF10) is commendable, despite of its high cement substitution, as it either shows higher compressive strength than other binary mixes (WSA10 and WSA30) or comparable to ternary mix WSA25SF5, despite of its low cement substitution. These results are, once again, attributed to the addition of a high percentage of very fine SF (10%) due to its better packing and filling abilities at early ages (7 days). Among all the mixes, the highest compressive strength at 7 days was demonstrated by the binary mix WSA20, while at 28 and 91 days by the ternary mix WSA33SF7.

In contrast to compressive strength, Figure 2b demonstrated a higher splitting tensile strength (STS) development for all concretes tested (binary and ternary) than that of CC. However, among binary and ternary mixes, their trends of STS development with respect to cement substitutions remained similar to that of compressive strength development. Moreover, similar to the compressive strength, the evolution of STS for WSA20 and WSA33SF7 was significantly higher as compared to CC and all other binary and ternary mixes. The only exception was at 7 days when WSA33SF7 exhibited higher STS than only CC. It is worth mentioning that the WSA20 concrete exhibited highest STS among all the concretes tested at all ages.

To summarize, the current findings demonstrated a decrease in compressive strength owing to a low (WSA10) or high (WSA30) cement replacement with WSA. Contrarily, a significant increase in both compressive and splitting tensile strength was obtained for 20% cement replacement with WSA (WSA20). Similar to binary concretes, the compressive strength of ternary concrete mixes decreased owing to a low (WSA25SF5) or high (WSA40SF10) cement replacements. However, a significant increase in both compressive and splitting strengths was observed for ternary concrete blend having 33% WSA jointly with 7% SF (WSA33SF7).

### 3.2. Comparison of the Water Absorption and Apparent Porosity of Control Concrete and Binary and Ternary Concrete Mixtures

**Figure 3:**
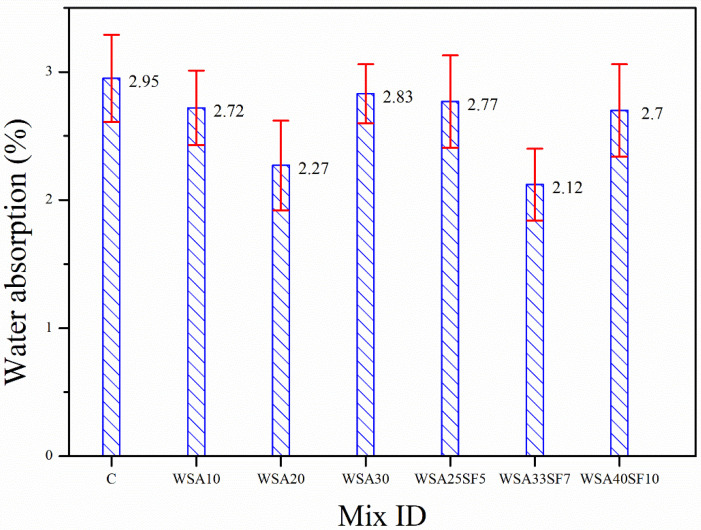
Comparison of the results for water absorption between the control and other concretes (binary and ternary mixes) after 91 days of standard curing.

**Figure 4:**
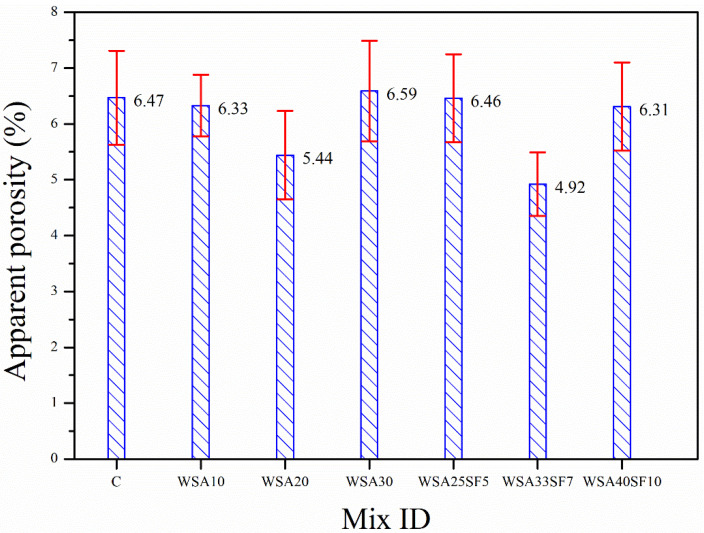
Comparison of the results for apparent porosity between control and other concretes (binary and ternary mixes) after 91 days of standard curing.

The comparison of the other important properties (WA and AP) of the binary and ternary concrete mixtures with that of the CC was also performed in addition to the strength characteristics. This is because the durability of the hardened concretes can be indirectly assessed based on the WA and AP values. The different trends of changes in the WA and AP according to various amounts of cement substitution with WSA alone (binary concretes) and WSA with SF (ternary concretes) are depicted in Figure 3 and Figure 4. It can be seen in Figure 3 that a lower WA compared to CC was exhibited by all binary and ternary concretes regardless of the amount of cement substitution, which is attributed to their lower AP values as compared to CC (Figure 4). A decrease in the WA of binary concrete mixtures with increasing percent substitution of cement with WSA was observed up to a certain replacement level (20%). Thus, a slightly higher WA was exhibited by binary concrete having 30% WSA when compared with other binary concretes having 10% or 20% WSA (Figure 3). These binary concretes also demonstrated a similar trend of AP (Figure 4). A slightly higher value of AP exhibited by concrete containing a high amount of WSA (30%) is probably due to a relatively slower rate of the pozzolanic reaction owing to replacement of high percentage of cement with WSA. On the contrary, ternary concretes having high cement substitutions with blends of WSA and SF demonstrated a remarkable decrease in WA and AP values. The results demonstrated that, despite similar cement substitutions (30%), the ternary mix WSA25SF5 exhibited slightly lower WA and AP to that of corresponding binary mix WSA30. However, contrary to this, the WA and AP of this ternary mix were slightly higher than the other binary mixes with 10% and 20% WSA. Increasing the cement replacement from 30% to 40% in ternary concrete (WSA33SF7) resulted in further decrease in WA and AP values. The results demonstrated that the ternary concrete WSA33SF7 with an even higher percent replacement of cement exhibited lower WA and AP than the ternary concrete WSA25SF5 and all binary concretes having relatively lower cement substitutions (10%, 20%, or 30%). This was because SF particles of very fine size leads to significant pore refinement in ternary concrete mixtures compared to WSA. The ternary concrete having a very high cement substitution of 50% (WS40SF10) yielded slightly higher WA and AP values when compared to those of WSA33SF7 with relatively low cement substitution (40%). This was due to potentially slower rates of pore refinement and pozzolanic reaction in WSA40SF10 concrete owing to high cement substitutions.

### 3.3. Evaluation of Compressive and Tensile Strength Correlation of Concrete by Prediction Models

Regardless of the type of mixture proportions, curing conditions, aging, or binder type and its content, the values of the experimental STS of concrete can be correlated with their corresponding compressive strength, mainly due to the existing consistency between their general trends of development with aging. In addition to the existing codes such as ACI 318 [51], ACI 363 [52], and CEB-FIP model code 1990 [53], various correlations between compressive and tensile strength were developed by researchers depending upon their specific experimental data of the curing and testing conditions, geometry of specimen, and the types of concrete [54,55,56,57,58,59,60]. Nevertheless, a consistent equation in general form [$f_{sp\;}=a\;\left(f_{c}^{\prime }\right){\;}^{b}$] is used for this correlation by all researchers, including the existing model codes. This equation presents $f_{sp\;}$ as the unknown STS of concrete to be predicted (MPa), whereas the compressive strength obtained directly from the experiments is represented by $f_{c}^{\prime }$ (MPa). In the equation, the parameters a and b are the constants that consider the dissimilarity of increasing rate between both mechanical properties. Based on different test results by researchers, the value of b varies, for example, as 0.67, 0.50, and 0.71 by the CEB-FIP model code [53], ACI 318 [51], and Kim et al. [57], respectively. The reason for dissimilarity in the b values arises since both ACI 318 and Kim et al. used specified and mean compressive strength, respectively, while the CEB-FIP model code used compressive strength associated with the specific characteristic compressive strength. Moreover, experimental results of 28 days were used in developing most of these correlations using Type-I cement for normal concrete subjected to standard moist curing at 20 °C. Despite consideration of the effect of various influencing factors (different binder types, curing, and aging) on the rate of both properties by some researchers [57], the effects of some other influencing factors such as the type of concrete using SCMs, geometry of specimen, and seasonal variations were overlooked. Having considered this important factor, it is desirable to evaluate suitability of existing correlations to predict the STS of concretes produced in this study containing various percentage of WSA alone (WSA10, WSA20, WSA30) and blends of WSA with SF (WSA25SF5, WSA33SF7, WSA40SF10).

**Figure 5:**
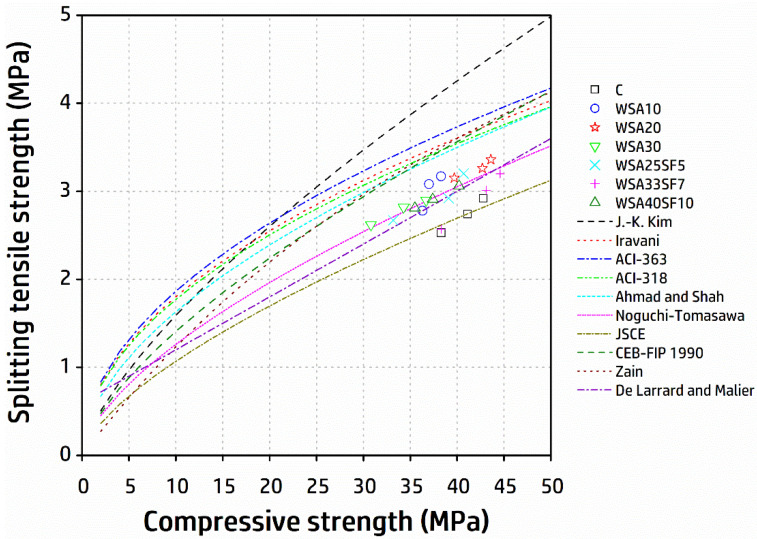
Comparison of the correlation between experimental compressive and tensile strengths for different concrete mixtures with existing prediction models.

Figure 5 depicts various existing correlations between compressive and tensile strengths of concrete. To evaluate the STS based on the experimental compressive strength, the current results of various binary and ternary concrete specimens along with CC were drawn with respect to 3, 7, and 28 days of aging and compared to existing models [51,52,53,54,55,56,57,58,59,60]. The experimental results of compressive strength were used to estimate the values of STS for the prediction models. With the exception of Noguchi-Tomosawa [58] and JSCE-2012 design codes [59], and De Larrard and Malier [60], all other existing models significantly overestimated STS, as shown in Figure 5. For the CC and WSA33SF7 at any known value of the compressive strength, a close match of the STS with that of the experimental compressive–tensile strength was predicted, regardless of aging, when using the JSCE-2012 model [$f_{sp\;}=0.23\;\left(f_{c}^{\prime }\right){\;}^{2/3}$]. The Noguchi–Tomosawa model [$f_{sp\;}=0.291\;\left(f_{c}^{\prime }\right){\;}^{0.637}$], on the other hand, resulted in a close match for the binary (WSA10, WSA20, WSA30) and other ternary (WSA25SF5, WSA40SF10) concretes. Similarly, a reasonably good estimate of the STS for any value of the compressive strength for these mixes was also predicted by the proposed model of De Larrard and Malier [$f_{sp\;}=0.6+0.06\;\left(f_{c}^{\prime }\right)$].

From these findings, it is revealed that the correlation between the compressive and splitting tensile strengths of concrete is not significantly influenced by the type of binder and aging. Kim et al. [57] also noted the same independency with respect to the cement type, aging, and curing temperature with no effect on the correlation of compressive and tensile strengths of concrete. The models proposed by either De Larrard and Malier [60] or Noguchi-Tomosawa [58], hence, are considered safe in estimating the STS of all the concrete mixtures studied with the exception of the CC and WSA33SF7 concrete. The JSCE model [59], however, accurately predicted STS of the CC and WSA33SF7 concrete. Furthermore, the JSCE model, with slight underestimation, satisfactorily predicted the STS of all the concrete tested. The underestimation of the STS with respect to the current experimental values is considered safe because it is used as the criterion of crack control.

### 3.4. SEM–EDS Analysis of Control (C), Binary (C/WSA), and Ternary (C/WSA/SF) Cementitious Pastes

**Figure 6:**
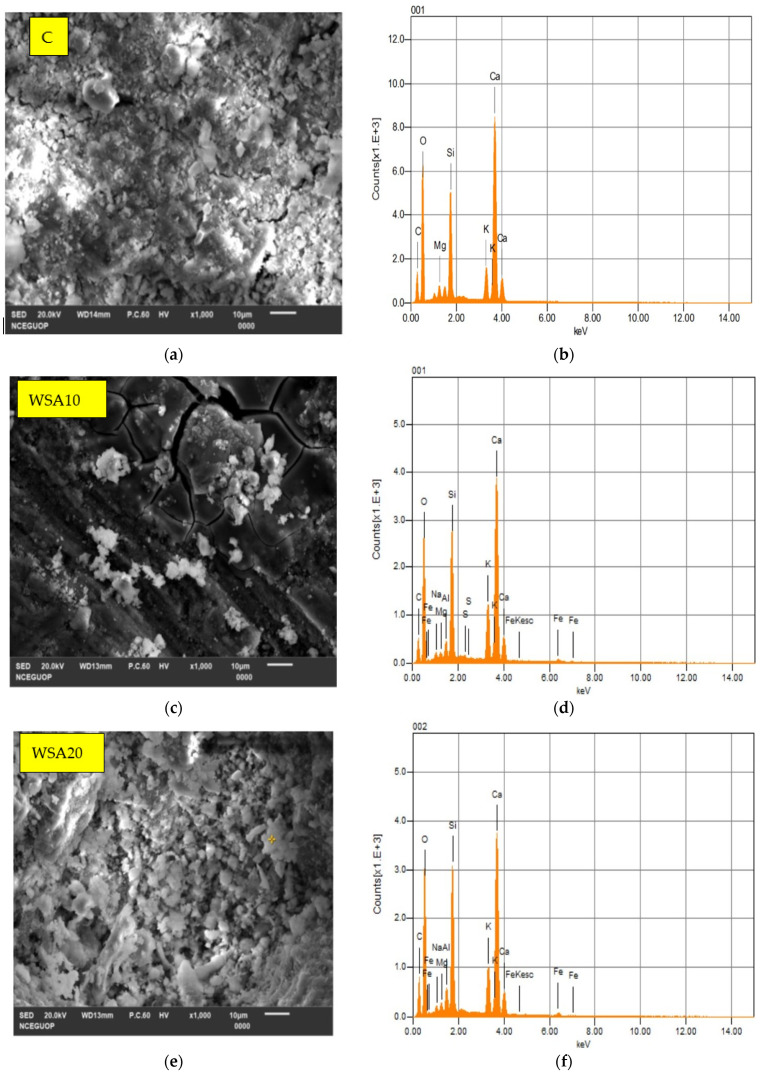

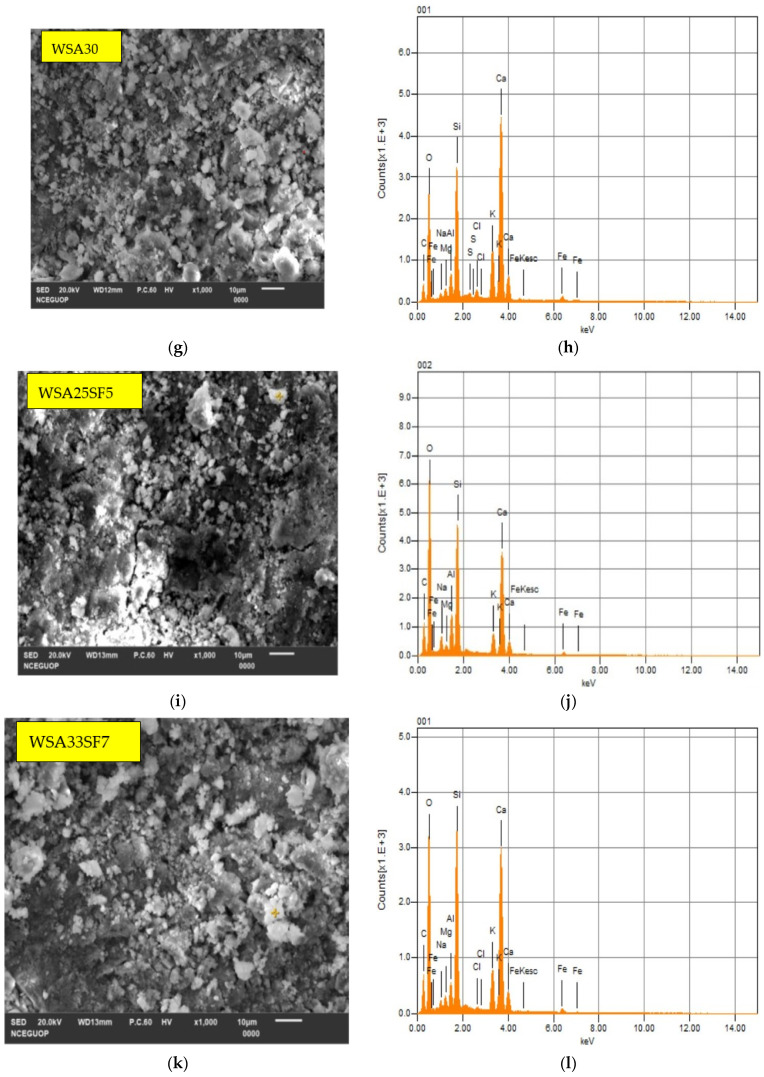

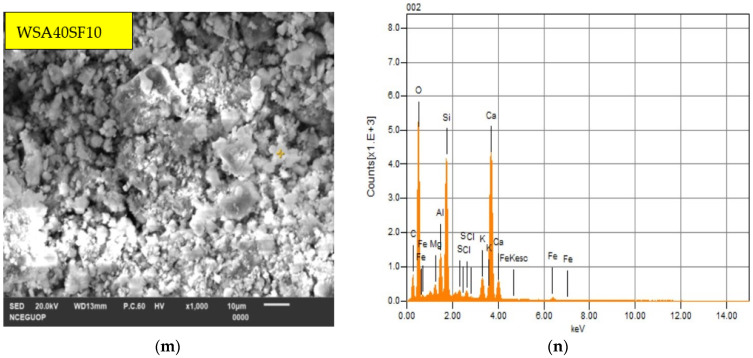
SEM–EDS spectrum of control, binary (C/WSA), and ternary (C/WSA/SF) paste samples (**a**,**b**) control, (**c**,**d**) WSA10, (**e**,**f**) WSA20, (**g**,**h**) WSA30, (**i**,**j**) WSA25SF5, (**k**,**l**) WSA33SF7, (**m**,**n**) WSA40SF10.

Figure 6 shows the results of SEM–EDS analysis for different mixes. As shown in this figure, the effects of WSA and SF on the microstructure of cementitious paste were examined through EDS analyses that were performed on SEM micrographs. The purpose of the EDS analysis with SEM was to study the crystal structure changes in C-H and C-S-H phases of paste matrix. Based on the computation from EDS analyses, a comparison of Ca/Si ratios among different mixes is presented in Table 6.

According to the findings of past researchers [61,62,63], the Ca/Si ratio for the C-S-H phase in paste sample ranges between 0.5 to 2.0, while 2.0 or higher for C-H phases. Among all mixes, both C-H and C-S-H phases of the control mix exhibited a highest Ca/Si ratio, at 3.30 and 1.93, respectively. As illustrated in Table 6, the lower Ca/Si ratio values of both the binary and ternary mixes is due to the substitution of cement with WSA and SF that led to decreased porosity of the paste matrix by forming high-density C-H and C-S-H phases in these mixes. Among binary mixes, WSA20 exhibited lowest Ca/Si ratio at 2.65 and 1.42 for C-H and C-S-H, respectively. Therefore, current results suggest a 20% replacement of cement with WSA without compromising the mechanical and microstructural performance of concrete. Moreover, all the ternary mixes showed even lower Ca/Si ratio as compared to binary mixes. A significant decrease in Ca/Si ratio in ternary mixes is attributed mainly to the fine, amorphous, and highly reactive nature of SF, which is used in ternary mixes in the presence of WSA. The addition of SF along with WSA results in the formation of additional C-S-H phases by utilizing C-H phases in the paste matrix. Furthermore, the addition of SF further enhances both the density of C-S-H and C-H phases and the compactness of cement paste. Among ternary mixes, the lowest Ca/Si ratio was observed for WSA33SF7, which indicates its improved microstructural properties due to the formation of high-density C-H (2.1) and C-S-H (0.91) phases. It is expected that such formation of high-density C-S-H and C-H phases would result in densification and refinement of the microstructure, leading to enhanced performance in practical engineering applications.

### 3.5. Fourier Transform Infrared (FTIR) Analysis of Cement Pastes

**Figure 7:**
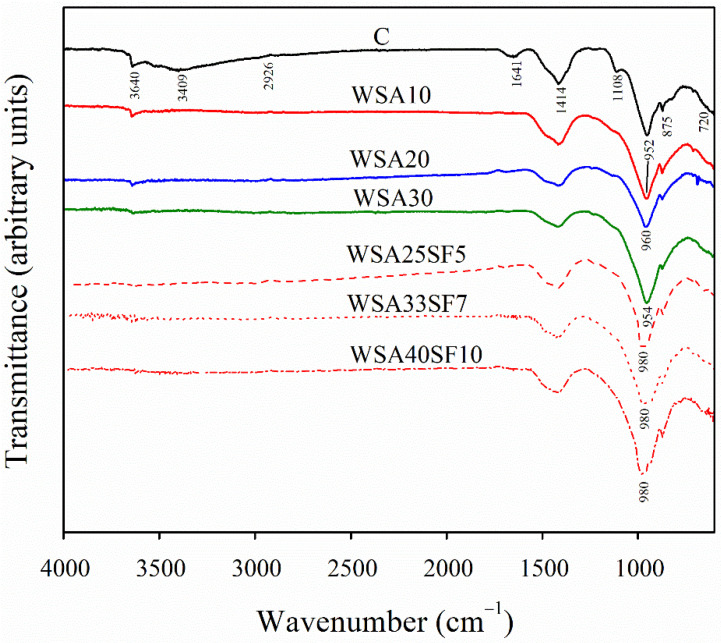
FTIR spectra of control, binary (C/WSA), and ternary (C/WSA/SF) paste samples after 91 days of curing.

As illustrated in Figure 7, all the paste samples show appearance of IR bands at the same location; however, their intensities differ. This is attributed to the formation of hydration products such as C-S-H and C-H [64]. The peaks from 900 to 1100 cm^−1^ are associated with vibrations of Si-O bands in C-S-H phase [65]. The IR bands show a higher relative intensity of the Si-O band in paste samples containing 20% WSA (WSA20). Further, the samples of ternary blends having WSA and SF showed better results than those of control and binary mixes. This shift in the Si-O band is associated with polymerization of silica. A slight shift (960 cm^−1^) was detected in WSA20. On the other hand, the ternary concrete samples with different percentages of SF (5%, 7%, and 10%) showed a broader and significant shift (980 cm^−1^). A shift toward a higher wavenumber in the spectrum of binary and ternary pastes suggests formation of a high amount of C-S-H gels. A large amount of C-S-H gels are produced from the nucleation sites provided by the fine particles of SF, as also observed earlier through SEM–EDS analysis. The development of high compressive strength in these mixes can be linked to the formation of a large amount of C-S-H gels. Moreover, the peaks at 720, 875, and 1415 cm^−1^ are associated with calcite formed as a result of carbonation [66].

In all the paste samples, the peak at 3645 cm^−1^ indicates the presence of free OH groups, which suggests the presence of the portlandite phase. The control sample exhibits a wider and more visible peak as compared to all other binary and ternary samples. However, this portlandite peak was reduced in all the binary mixes, which indicates the extent of portlandite consumption caused by the presence of amorphous silica in WSA. Interestingly, in ternary mixes containing WSA and SF, the peak remained very small or almost disappeared, indicating a high pozzolanic reactivity, which consequently results in greater consumption of C-H. This leads to formation of more C-S-H gels in these mixes [67]. As discussed in the preceding section, similar evidence of this large amount of C-S-H gels in these mixes was also noticed in SEM–EDS analyses.

### 3.6. Nitrogen Adsorption Results (Surface Area and Pore Structure of Cement Pastes)

**Figure 8:**
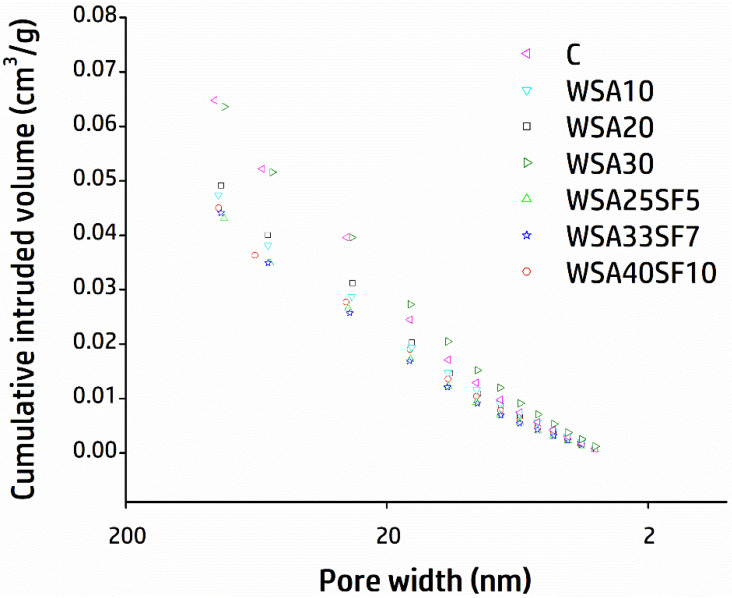
Comparison of the cumulative nitrogen intrusion volumes versus pore widths for control, binary (C/WSA), and ternary (C/WSA/SF) paste samples after 91 days of curing.

Figure 8 shows the comparison of cumulative nitrogen intrusion volume with respect to pore width for different paste samples after curing for 91 days. The results demonstrate lesser pore volume for ternary mixes having WSA together with SF as compared to control as well as binary mixes containing WSA. The relatively lesser pore volume of ternary mixes indicates formation of dense pore structure due to accelerated pozzolanic reactivity caused by the highly reactive SF. The least amount of nitrogen intrusion volume (0.043 cm^3^/g) was observed for a ternary mix with 40% cement substitution (WSA33SF7) followed by WSA25SF5 (0.045 cm^3^/g), WSA40SF10 (0.046 cm^3^/g), WSA10 (0.048 cm^3^/g), WSA20 (0.049 cm^3^/g), control (0.050 cm^3^/g), and WSA30 (0.051 cm^3^/g). The binary mixes with lower percent of WSA (WSA10 and WSA20) exhibited lesser nitrogen intrusion volume as compared to control, however, the maximum nitrogen intrusion volume was observed for WSA30. This is most obviously due to reduced pozzolanic reactivity when a high percent of cement replaced with WSA, which consequently had led to increased porosity and vascularity in the paste matrix.

**Figure 9:**
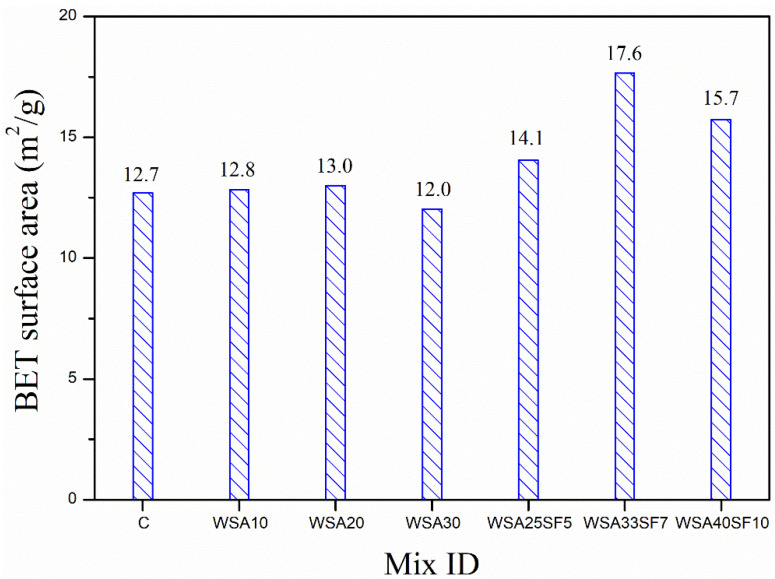
Comparison of BET surface area between control, binary (C/WSA) and ternary (C/WSA/SF) paste samples after 91 days of curing.

A comparison of Brunauer–Emmett–Teller (BET) surface areas between control and other binary and ternary mixes is presented in Figure 9. All ternary mixes exhibited higher BET surface areas as compared to those of binary and CC. Similar to the least amount of nitrogen intruded volume, WSA33SF7 demonstrated a larger BET surface area (17.6 m^2^/g) as compared to all other mixes. An increased BET surface area of the ternary mix indicates its improved and denser microstructure of C-S-H gels [68,69]. As described in the preceding section, SEM analysis with EDS demonstrated a dense cementitious matrix with few pores. In general, BET surface area increased with increasing percent of WSA and SF in ternary mixes, except for WSA40SF10 (15.7 m^2^/g), which showed a slight reduction that could be due to a high amount of cement substitution leading to a large amount of unreacted WSA in the mixture [70]. Similar to ternary mixes, binary mixes, especially those containing relatively low percentage of WSA such as WSA10 (12.8 m^2^/g) or WSA20 (13.0 m^2^/g), also showed slightly larger BET surface areas than that of control (12.7 m^2^/g), which ultimately had led to their improved microstructure of C-S-H gel. However, contrary to this, BET surface area decreased with further increase in percent of WSA (more than 20%) as was observed for WSA30 (12.0 m^2^/g). This clearly indicates the presence of unreacted WSA that had caused the hydration products to jam the pores and form a porous paste matrix. These results suggest a restricted use of WSA in binary mixes by not more than 20% when used as a sole substitute of cement.

### 3.7. Thermogravimetric Analysis (TGA) of Cement Pastes

**Figure 10:**
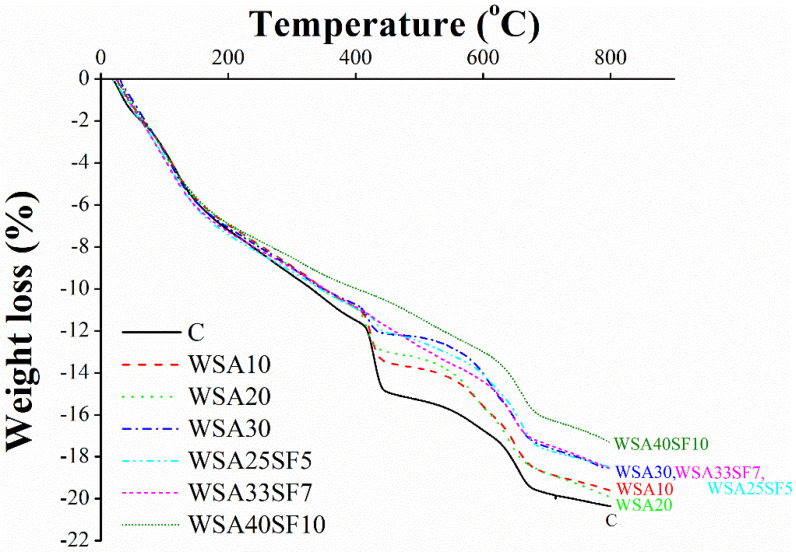
Comparison of thermogravimetric analysis of 91-day cured paste samples among control, binary (C/WSA), and ternary (C/WSA/SF) paste samples after 91 days of curing.

The thermal decomposition of 91-day cured cement paste samples of control, binary, and ternary mixtures was performed by TGA to evaluate the effect of WSA and SF on the amount of C-H (%), which occurred due to the weight loss between 400 and 500 °C [71,72]. Based on the TGA results, a comparison of weight loss (%) with respect to temperature is presented between control, binary, and ternary mixes (Figure 10). Consequently, using the TGA results, the amount of C-H for different mixes and their normalized C-H (%) values with respect to OPC was calculated, as listed in Table 7. The normalized C-H values for each mix were calculated by dividing their C-H values with their respective OPC (%) content.

From Table 7, it can be seen that the maximum C-H content exists in control sample and a gradual decrease in C-H phase occurred in binary mixes with increasing percent substitution of cement with WSA. The gradual decrease in C-H content in binary mixes is obviously due to their decreasing cement content and partly because of the pozzolanic reactivity caused by WSA due to the formation of hydration products as a result of C-H consumption. However, as compared to binary mixes, a significant decrease in C-H phase occurred in ternary mixes, which was due to the simultaneous effects of high reactivity of SF along with pozzolanic reaction caused by WSA. This would ultimately lead to significantly high densification and compactness of C-S-H gel microstructure for ternary mixes as compared to binary and control mixes.

Similar to C-H, it can be further seen from Table 7 that the normalized C-H values of all the binary and ternary mixes are lower as compared to control. However, in comparison to ternary, all the binary mixes exhibited relatively higher normalized C-H values, which demonstrated their lesser pozzolanic reactivity. In fact, the same was also evidenced earlier through XRD, SEM–EDS, and FTIR analyses. Moreover, the reason for relatively lower normalized C-H in ternary mixes, despite their large volume substitution of cement, is mainly due to the high reactivity of SF jointly with pozzolanic WSA, which ultimately causes a seeding effect to produce more C-S-H gel. These important findings with clear scientific proofs may justify the effectiveness of using both WSA and SF jointly as a high-volume replacement of cement for the production of a strong, durable, and sustainable concrete.

### 3.8. Global Warming Potential

#### 3.8.1. Comparison of CO_2_-eq for Unit Concrete Production among Control, Binary, and Ternary Concrete Mixes

**Figure 11:**
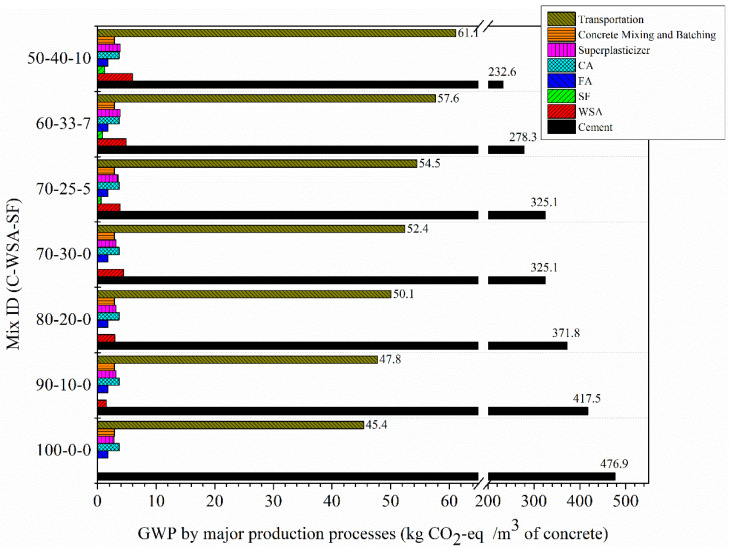
Comparison of GWP distribution by concrete ingredient and phase between control, binary (C/WSA), and ternary (C/WSA/SF) concrete mixtures.

Figure 11 shows the comparison of estimated GWP (kg CO_2_-eq per unit volume concrete) between control and other binary as well as ternary concrete mixtures. In this figure, the distribution of CO_2_-eq for each concrete mixture is illustrated by its ingredients and major production processes. The value of CO_2_-eq for all concrete mixtures was calculated by using the green LCA tool according to the data listed in Table 4 and Table 5. Subsequently, the total emission for each concrete mixture was calculated by adding its direct and supply chain emissions associated with the quarrying, production, and transportation processes that occur within a systems’ boundary.

It can be seen in Figure 11 that among all the different ingredients used in unit concrete production, cement is responsible for maximum CO_2_ emission for all the mixtures. Following cement production, transportation of the raw materials and their products represents the second-highest source of CO_2_ emissions, which varies between 8.5% and 19.5%. Most importantly, the CO_2_ emissions associated with noncementitious materials are very low and remained almost same for all concrete mixes. According to the calculations, the SCMs (WSA and SF) constituted only 0.3% to 1.4% of total calculated CO_2_ emissions. Similarly, concrete batching and mixing account for very small percentages, between 0.4% and 0.5%.

According to the comparison, the CC exhibited the highest GWP at 533 kg CO_2_-eq/m^3^ among all mixes (Figure 11). On the other hand, the ternary concrete mix containing WSA together with SF (WSA40SF10) as 50% cement substitution possessed lowest GWP at 313 kg CO_2_-eq/m^3^. Generally, by decreasing the amount of Portland cement in concrete mixes and increasing the amount of SCMs, the carbon footprint for cement production decreased from 89% for CC to 44% for WSA40SF10.

#### 3.8.2. Comparison of Normalized CO_2_-eq for Unit Concrete Production per Unit Strength (MPa) among Control, Binary, and Ternary Concrete Mixes

**Figure 12:**
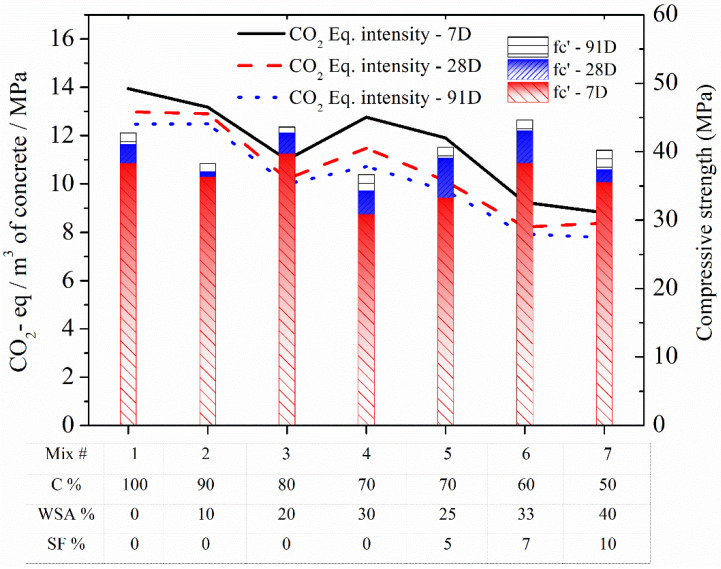
Comparison of normalized GWPs as kg CO_2_-eq with respect to strength among control, binary (C/WSA), and ternary (C/WSA/SF) concrete mixtures.

Figure 12 shows the comparison of normalized values of GWPs as CO_2_-eq/m^3^/MPa among all the mixes, including control, binary, and ternary (Mix #1 to 7). As illustrated in Figure 12, the normalized values of CO_2_-eq/m^3^/MPa for all the mixes were drawn with respect to their compressive strength and aging (7, 28, and 91 days). The CO_2_-eq intensity of different mixes is used as a measure to evaluate their important impact on compressive strength of concrete and associated GWP per unit concrete volume and strength [73].

It can be seen in Figure 12 that each individual mix, in general, showed a gradual decreasing CO_2_-eq/m^3^/MPa trend due to increasing compressive strength with aging. Moreover, it can be further noted that the amount of CO_2_-eq/m^3^/MPa decreases with decreasing quantity of cement in all the binary and ternary mixtures. The only exception is the binary mix WSA30, where it slightly increased as compared to WSA20. This is due to drop of strength in WSA30 because of the high amount of cement substitution (30%) in this mix as compared to WSA20. A sharp decrease in CO_2_-eq/m^3^/MPa in a binary (WSA20) and ternary mix (WSA33SF7) is due to their high compressive strengths as compared to other mixes.

The current results dictate the possibility of lowering CO_2_-eq intensities by replacing cement with only one type of SCM (WSA) or blends of SCMs (WSA with SF). According to the current findings, the intensities of CO_2_-eq improved significantly in ternary blends as compared to binary, without compromising any strength potentials. For instance, despite of almost similar compressive strength of binary WSA20 (43.6 MPa) and ternary WSA33SF7 (44.6) mixes at 91 days, CO_2_-eq intensity decreased to 7.9 kg/m^3^/MPa in ternary mix (mix #6 having 60% cement) from 10 kg/m^3^/MPa in binary mix (mix #3 having 80% cement). Furthermore, the addition of higher amounts of WSA (40%) and SF (10%) in ternary mix (mix #7 having 50% cement) causes a further reduction in CO_2_-eq intensity as 7.8 kg/m^3^/MPa as compared to 12.5 kg/m^3^/MPa in binary mix (mix #2 having 90% cement). This might be due to slightly higher 91-day strength of ternary mix (40.2 MPa) as compared to binary mix (38.3 MPa). These findings are equally applicable for these mixes at other ages as well, such as 7 and 28 days. Consequently, these results suggest that the intensities of CO_2_-eq can be reduced without compromising the strength of concrete by optimizing concrete mixes with appropriate amount of cement replacement and selection of a suitable type of SCMs with their correctly chosen or tested blend percentages.

## 4. Conclusions

This study investigated the use of high-volume WSA and its blend with SF as a partial substitute of cement for the development of high-performance and sustainable concrete. Besides control (100% cement), several other concrete mixtures were prepared by partially substituting cement with only WSA as binary system (10%, 20%, and 30%) and WSA together with SF as ternary system (25%/5%, 33%/7%, and 40%/10%). Subsequently, the influence of adding WSA and WSA with SF on the hardened mechanical properties (compressive and tensile strengths, WA, and AP) was assessed and compared to those of CC. Finally, paste samples were prepared for all mixes to examine their microstructures and pore structures using SEM–EDS, FTIR, TGA, and N_2_ adsorption techniques to scientifically understand the impact of adding WSA and SF on the resulting paste matrix. At the end, GWP as kg CO_2_-eq per unit volume of concrete and kg CO_2_-eq per unit volume of concrete/MPa were also calculated using the LCA tool and compared among different mixes used in this study.

The main findings of this study are summarized as follows:

The current findings demonstrated a decrease in strength of binary concrete corresponding to their relatively low (WSA10) and high (WSA30) cement substitution rates, while a significant increase in strength was observed for moderate substitution rate of cement (20%) for binary concrete WSA20. In a very similar manner, the ternary mixes also showed a decreasing trend of strengths both at low (WSA25SF5) and high (WSA40SF10) blends of cement substitution, and the significant increase in strength was obtained for the moderate ternary blend of 33% WSA and 7% SF (WSA33SF7). Moreover, these increased strengths of WSA20 and WSA33SF7 were validated by their relatively lower apparent porosity and water absorption values among all mixes.

A correlation between experimental compressive and tensile strengths showed close agreement to models proposed by Noguchi–Tomosawa and De Larrard and Malier. Based on the results of current findings, it is recommended to use these models to properly estimate the tensile strength of tested concretes containing WSA alone or WSA jointly with SF, except the CC and WSA33SF7, as the JSCE model demonstrated a close agreement for these two concrete mixes. In addition, the JSCE model safely predicts the tensile strength of other binary and ternary concrete mixes with their slightly underestimated values.

Analysis of SEM–EDS data reveals that the incorporation of WSA as 20% replacement of cement (WSA20) leads to the densification of the paste matrix by decreasing the Ca/Si ratio in both the C-S-H and C-H phases. Furthermore, adding a 7% SF jointly with 33% WSA in the ternary mix (WSA33SF7) resulted in the lowest Ca/Si ratio among all the concrete mixtures tested. These findings suggest better refinement of microstructure for these mixes, which ultimately would lead to improvement of their engineering performance.

A visible shift in Si-O band was observed through FTIR analysis for almost all the binary and ternary mixes. Comparatively, the shift was more pronounced in all ternary mixes containing WSA together with SF, which clearly demonstrates the presence of high levels of C-S-H gels. Moreover, the portlandite peaks (3641–3644 cm^−1^) were also significantly smaller in all ternary mixes as compared to binary mixes. This, consequently, suggests an improved pozzolanic reactivity and the formation of more C-S-H gels that ultimately leads to a more refined and denser microstructure.

Similar to FTIR analysis, a densification of the paste matrix and refinement of the pore structure also suggested by the results of N_2_ adsorption tests was due to the decrease in intruded pore volume and an increase in BET surface area, especially for mixes WSA20, WSA25SF5, and WSA33SF7. However, other mixes such as those containing a large amount of WSA (WSA30 and WSA40SF10) showed a smaller surface area and more intruded pore volume. This could be possibly due to the presence of unreacted WSA in these mixes that might have caused high porosity and vascularity in their paste matrices.

As a matter of further validation of these findings, TGA results also showed a reduction in the portlandite phase of binary and ternary mixes. This occurred especially in those binary mixes that contained high doses of WSA (WSA20 and WSA30), partly because of a lesser amount of cement in these mixes and due to the pozzolanic reactivity of amorphous silica present in WSA. A further reduction in the proportion of portlandite phase occurred in ternary mixes due to very fine and amorphous silica present in both WSA and SF, which would ultimately consume the C-H phase to generate additional C-S-H phases and lead to densification of the paste matrix.

The GWP per unit volume of concrete (CO_2_-eq/m^3^) mixes decreased with decreasing amount of Portland cement simultaneously with an increase in amounts of SCMs. The highest GWP of 533 was generated by the control mixture (100% OPC) while the least GWP, only 313, was produced by the ternary concrete mixture (WSA40SF10) having 40% WSA jointly with 10% SF and only 50% OPC.

Regardless of aging, all the binary and ternary concrete mixes containing SCMs (WSA or WSA with SF) exhibited lower CO_2_-eq intensities as compared to CC, with the only exception of a binary mix having 10% WSA that showed almost identical CO_2_-eq intensities to that of control at later ages of 28 and 91 days. Furthermore, as compared to binary, ternary mixes having WSA together with SF showed good potential for further reducing the normalized CO_2_-eq intensities/MPa at all ages (7, 28, and 91 days). The ternary mixes containing highest percentages of SCMs, at 40% (WSA33SF7) and 50% (WSA40SF10), resulted in lower CO_2_-eq intensity/MPa as compared to that of CC, regardless of aging. Consequently, it may be safe concluding that the efforts for using larger amounts of regionally available SCMs can have an important positive effect on producing green concretes together with reduced GWP without compromising any strength potentials.

## Figures and Tables

**Table 1:** Physical and chemical properties of cement, WSA, and SF.

	OPC	WSA	SF
	Physical properties		
Specific gravity (kg/m^3^)	3.20	2.21	2.23
Blain fineness (m^2^/g)	0.344	-	21.5
	Chemical properties (oxides, % by weight)		
SiO_2_	21.3	65.1	93.3
Al_2_O_3_	5.56	9.10	-
Fe_2_O_3_	3.24	2.67	0.58
SiO_2_ + Al_2_O_3_ + Fe_2_O_3_ *	-	76.87	-
CaO	63.4	5.90	1.82
MgO	0.93	1.11	0.28
Na_2_O	0.13	0.40	0.19
K_2_O	0.62	10.5	0.88
SO_3_	2.25	1.13	-
LOI **	2.41	4.03	2.25
	Mineral composition (%) ***		
C_2_S C_3_S C_3_A C_4_AF	47.9 24.7 8.76 9.86	- - - -	- - - -

* ASTM C618-15; ** LOI = loss on ignition; *** data from local cement manufacturer.

**Table 2:** Sieve analysis of aggregates (ASTM C136).

Sieve #	Sieve Size (mm)	Weight Retained (%)	Cumulative Passing (%)	Cumulative Retained (%)
Coarse aggregate (CA)				
1 inch	25	0	100	0
3/4 inch	19	0	100	0
1/2 inch	12.5	50.5	50	50
3/8 inch	9.5	26.1	23	77
No. 4	4.75	23.4	0	100
Fine aggregate (FA)				
3/8 inch	9.5	0	100	0
No. 4	4.75	0	100	0
No. 8	2.36	4.54	95.46	4.54
No. 16	1.18	16.1	79.33	20.67
No. 30	0.600	30.9	48.40	51.60
No. 50	0.300	26.4	22.02	77.98
No. 100	0.150	21.3	0.67	99.33
Pan	-	0.67	-	-
Fineness Modulus of FA (**FM**) = (0 + 4.54 + 20.67 + 51.6 + 77.98 + 99.33)/100 = **2.54**				

**Table 3:** Mixture proportions for control, binary, and ternary concretes (w/b = 0.35; a/b = 3.37; s/a = 0.40).

Mix ID (Total # of Specimens for Each = 18 and Cured for 7, 28, 91 Days)	W (kg/m^3^)	Binder, b (kg/m^3^)			Aggregates, a (kg/m^3^)			Superplasticizer (% of b)	Slump (mm)	
		C	WSA	SF	FA (S)	CA 10 mm	CA 20 mm			
Control concrete (CC)	160	457	-	-	617	462	462	1	120 ± 30	
Concrete containing 10% WSA (WSA10)		411	46	-				1		
Concrete containing 20% WSA (WSA20)		366	91	-				1		
Concrete containing 30% WSA (WSA30)		320	137	-				1.1		
Concrete containing 25% WSA and 5% SF (WSA25SF5)		320	114	23				1.2		
Concrete containing 33% WSA and 7% SF (WSA33SF7)		274	151	32				1.3		
Concrete containing 40% WSA and 10% SF (WSA40SF10)		229	182	46				1.4		

**Table 4:** Assumptions used in LCA calculations.

User Input Data:	Type of Material	
**Ordinary Portland cement (OPC)**	ASTM Type I	
SCMs	Silica fume (SF), Wheat straw ash (WSA)	
Admixture	Superplasticizer	
**Electricity grid mix for:**	**Location**	
Cement supplier	Cherat, Pak	
Fine aggregates supplier	Larunspur, Pak	
Coarse aggregates supplier	Taxila, Pak	
Gypsum supplier	Dera Ismael Khan, Pak	
SF supplier	Karachi, Pak	
Wheat straw ash collection	Punjab, Pak	
**Transportation details for:**	**Mode**	**Distance (km)**
Cement raw materials to cement plant	Truck Class 8b	5
Gypsum to cement plant	Truck Class 8b	300
Cement to concrete plant	Truck Class 8b	200
Fine aggregates to concrete plant	Truck Class 8b	200
Coarse aggregates to concrete plant	Truck Class 8b	100
Admixture to concrete plant	Truck Class 2	1000
WSA to concrete plant	Truck Class 8b	200
SF to concrete plant	Truck Class 8b	1200
**Technology options for:**	**Type of technology selected**	
Cement raw materials prehomogenization	Dry, raw storing, preblending	
Cement raw materials grinding	Dry, raw grinding, tube mill	
Cement raw meal blending/homogenization	Dry, raw meal blending, storage	
Clinker pyroprocessing	Preheater/precalciner kiln with US average kiln fuel mix	
Clinker cooling	Reciprocating grate cooler (modern)	
Cement finish milling/grinding/blending	Tube mill	
Cement PM control technology	ESP	
Conveying within the cement plant	Screw pump	20 m between process stations
Concrete batching plant loading/mixing	Mixer loading (central mix)	
Concrete batching plant PM control	Fabric filter	

**Table 5:** Electricity grid mix percentage for Pakistan [50].

User Input Data:	National Grid (%)
Coal	12
Natural gas	29
Fuel oil	20
Pet coke	-
Nuclear	2.5
Hydropower	34
Biomass	0.5
Geothermal	-
Solar	1
Wind	1

**Table 6:** Ca/Si atomic ratio from EDS analysis of control and mixes containing different percentages of WSA alone and WSA with SF.

Mix ID	Ca/Si Atomic Ratio	
	C-S-H Phase	C-H Phase
CC	1.93	3.3
WSA10	1.63	3.1
WSA20	1.42	2.65
WSA30	1.6	2.94
WSA25SF5	1.05	2.16
WSA33SF7	0.91	2.1
WSA40SF10	1.24	2.3

**Table 7:** Actual amount of calcium hydroxide (C-H) and normalized with respect to the percent content of cement in each mix (C-H/OPC) after 91 days of cement hydration.

Mix ID	C-H (%)	C-H/OPC (%)
CC	18.5	18.5
WSA10	13.87	15.4
WSA20	11.28	14.1
WSA30	8.85	12.6
WSA25SF5	7.47	10.7
WSA33SF7	7.26	12.1
WSA40SF10	6.44	12.9

## Data Availability

All the data utilized in current research are available upon a reasonable request from the corresponding author.
